# Repeated Nitrous Oxide Exposure Exerts Antidepressant-Like Effects Through Neuronal Nitric Oxide Synthase Activation in the Medial Prefrontal Cortex

**DOI:** 10.3389/fpsyt.2020.00837

**Published:** 2020-09-03

**Authors:** Wei Liu, Qian Li, Binglu Ye, Hang Cao, Fuyi Shen, Zhendong Xu, Weijia Du, Fei Guo, Jinqi Liu, Tianyu Li, Bing Zhang, Zhiqiang Liu

**Affiliations:** ^1^Department of Anesthesiology, Shanghai First Maternity and Infant Hospital, Tongji University School of Medicine, Shanghai, China; ^2^Key Laboratory of Receptor Research, Shanghai Institute of Materia Medica, Chinese Academy of Sciences, Shanghai, China; ^3^The MacDuffie School, Granby, MA, United States; ^4^Clinical and Translational Research Center, Shanghai First Maternity and Infant Hospital, Tongji University School of Medicine, Shanghai, China; ^5^Anesthesia and Brain Function Research Institute, Tongji University School of Medicine, Shanghai, China

**Keywords:** nitrous oxide (N_2_O), depression, mPFC, burst firing, neuronal nitric oxide synthase (nNOS), BDNF

## Abstract

Clinical studies have demonstrated that exposure to the inhalational general anesthetic nitrous oxide (N_2_O) produces antidepressant effects in depressed patients. However, the mechanisms underlying the antidepressant effects of N_2_O remain largely unknown. Neuronal nitric oxide synthase (nNOS)–mediated nitric oxide (NO) synthesis is essential for brain function and underlies the molecular mechanisms of many neuromodulators. We hypothesized that activation of the nNOS/NO pathway in the medial prefrontal cortex (mPFC) might mediate the antidepressant effects of N_2_O. In this study, we revealed that repeated N_2_O exposure produced antidepressant-like responses in mice. Our mechanistic exploration showed that repeated N_2_O exposure increased burst firing activity and that the expression levels of BDNF with nNOS activation were dependent in the mPFC. In particular, the antidepressant-like effects of N_2_O were also antagonized by local nNOS inhibition in the mPFC. In summary, our results indicated that N_2_O exposure enhances BDNF expression levels and burst firing rates in an nNOS activation dependent manner, which might underlie the pharmacological mechanism of the antidepressant-like effects of N_2_O exposure. The present study appears to provide further mechanistic evidence supporting the antidepressant effects of N_2_O.

## Introduction

Major depressive disorder (MDD) is one of the most severe mental disorders in the world and accounts for most of the nonfatal burden of mental and substance use disorders (1). The currently the available antidepressants mostly target the monoaminergic system, but these classic antidepressants are associated with low remission rates and a lag in the onset of antidepressant action obtained (2–4), highlighting an urgent and clear need for identifying a more efficacious and faster-acting therapeutic agents.

In recent decades, studies on the antidepressant effects of anesthetics have attracted considerable attention. Recent studies have suggested that some anesthetics show promise as therapeutics against MDD. Ketamine has attracted keen interest due to its remarkably rapid and sustained antidepressant effects (5–7). However, when administrated as a single intravenous dose, ketamine is associated with some unwanted side effects, including dissociation, headache, dizziness, elevated blood pressure, and blurred vision (8). Thus, the safety concerns of ketamine should be carefully considered, particularly after repeated dosing.

Nitrous oxide (N_2_O) is a widely used inhalational general anesthetic (9). Recent clinical studies have shown that N_2_O exerts rapid and marked antidepressant effects in patients with treatment-resistant major depression (TRMD), and the improvements can last for a full week (10). Importantly, compared with ketamine, N_2_O does not have psychotomimetic or cognitive side effects (11). Hence, N_2_O might be an attractive alternative for the development of mood disorder therapeutic. Because the improvements in TRMD obtained with N_2_O are associated with relatively few safety concerns, studies of the mechanism of the N_2_O-induced antidepressant effects will support a better understanding of the pharmacological treatment of depression.

N_2_O influences brain functions through multiple mechanisms of action, and this agent is commonly used in dentistry and obstetrics because it is an effective analgesic and anxiolytic agent. The anxiolytic effect might involve the activation of GABA_A_ receptors through the benzodiazepine-binding site (12). N_2_O is also an antagonist of the N-methyl-D-aspartate (NMDA) receptor, which is a therapeutic target that might exert antidepressant effects (10, 11) similar to those obtained with ketamine. In addition, this agent has a wide range of other potential therapeutic targets, but the precise mechanism underlying the antidepressant effects of N_2_O has been less well addressed and might involve other molecular and receptor systems.

Nitric oxide (NO), a widespread signaling molecule that can be regulated by N_2_O, has diverse biological effects in the central nervous system (CNS) (13, 14). However, it remains unclear whether an increase in NO as a result of N_2_O is part of the antidepressant mechanism of N_2_O. Neuronal nitric oxide synthase (nNOS) is the key enzyme that mediates the NO signal transduction pathway in the brain (15), this enzyme participates in the regulation of learning, memory, and pathological conditions (16–19). Many shreds of evidence suggest that an imbalance of nNOS activity in the CNS implicated in the pathophysiology of MDD, and the nNOS system is proposed to be a potential therapeutic target for MDD (20). Thus, we hypothesized that the antidepressant effects of N_2_O might be mediated by the activation of the nNOS/NO pathway in the mPFC.

In this study, we observed antidepressant-like effects in mice after repeated N_2_O exposure. To address the underlying mechanism, we investigated the neuronal activity in the mPFC and further identified the functional roles of nNOS in the antidepressant-like effects of N_2_O.

## Materials and Methods

### Animals

Adult male CD-1 mice [weighing 25–35 g, postnatal day (PD) 42] were used in our experiments. The animals were housed in a limited-access rodent facility with four to five mice per cage in a room with a 12-h light/12-h dark cycle (cage size: 320 × 210 × 160 millimeters, lights on from 7:00 a.m. to 7:00 p.m.) and a constant temperature (22 ± 2°C). Sterilized drinking water and standard food were provided ad libitum. Before the experiments, the mice were habituated to these conditions for 2–3 days. In all experiments, animals were divided randomly into groups. Briefly, we numbered all animals and used Excel’s random number generator to randomize the animals to groups. All the animal studies and experimental procedures were approved by the Animal Care Committees of the Shanghai Institute of Materia Medica, Chinese Academy of Sciences, and the experiments were performed in accordance with EU Directive 2010/63/EU on the protection of animals used for scientific purposes.

### Stereotactic Surgery and Local Infusion

Adult CD-1 mice (aged 6–8 weeks) were anesthetized with pentobarbital sodium (80 mg/kg, i.p.) and fixed on an animal stereotaxic frame (RWD, 68016). Two 29-gauge guide cannulas were bilaterally placed 1 mm above the mPFC (anterior/posterior (A/P): +2.10 mm; medial/lateral (M/L): ± 0.35 mm; dorsal/ventral (D/V): -2.0 mm; **Supplementary Figure 3A**). A 39-gauge needle with a plastic cap on top was inserted into the guide cannula to prevent clogging during the recovery period. The cannulas were fixed to the skull using dental cement. After surgery, the mice were then allowed to recover for at least 1 week before the subsequent experiments. Drugs were microinjected with a 39-gauge injector cannula that was inserted into the guide cannula. One microliter of drug was infused (0.1 µl/min, 5 min total) into each side with a microsyringe. The injector cannulas were kept in place for an additional 5 min to minimize spread of the drug along the injection track. Finally, to confirm the accurate drug injection site and the spreading region of injected fluid, we have injected an equal volume of Chicago Sky Blue solution (2M NaCl and 0.5% Chicago Sky Blue) at the same coordinates in the mPFC (A/P: +2.10 mm; M/L: ± 0.35 mm; D/V: -3.0 mm). After behavioral tests, mice were sacrificed and each brain was sliced to examine the injection sites. Data were excluded where the injection site and the diffusion region were not located in the prelimbic subregion (PrL) of the mPFC.

### Drugs and Drug Administration

Mice were randomly divided into four groups. (i) Mice in the control group were exposed to air in the chamber to exclude the effects of gas exposures. In the vehicle (ii) and experimental *groups* (iii), mice were exposed to the 50% N_2_ + 50% O_2_ mixture and 50% N_2_O + 50% O_2_ mixture for one 2-h session per day for three consecutive days, respectively. The mice in the positive control (iv) group were exposed to ketamine (20 mg/kg) 24 h before behavioral test. The dose of 20 mg/kg is an available concentration to elicit antidepressant effects in rodents that was confirmed by previous reports (21, 22). For drug administration, the mice were temporarily placed in a transparent airtight chamber (35 cm × 25 cm × 15 cm) at room temperature for 10 min of acclimatization before the gas exposure sessions. The concentration of N_2_O was monitored in real time. The gas flow was maintained at 2–3 L/min. At the end of the drug administration period, the mice were returned to their home cages, and 24 h after drug administration, the behavioral experiments of the mice were tested.

For local mPFC administration, the mice were fixed with gauze and allowed to recover for 1 week. The intracerebral drug deliveries were given in awake animals. *The* NOS inhibitor L-N^G^-nitroarginine methyl ester (L-NAME, Abcam, ab120136) was dissolved in saline to a concentration of 10 μg/μL. Local L-NAME administration was performed 30 min prior to gas exposure. L-NAME was infused into the mPFC (0.1 µl/min, 5 min total).

For the electrophysiological experiments, L-NAME (20 mg/kg) was dissolved in saline and administered to the mice 30 min before every gas exposure through an intraperitoneal injection (i.p.).

### Forced Swim Test (FST)

The apparatus used for the FST was a transparent plastic cylinder with size of 35 cm height × 10 cm diameter. During the FST, mice were individually placed in a cylinder of water with the depth of 20–26 cm to prevent the mice from touching the bottom with their limbs. The behavior tests were performed 24 h after the final N_2_O exposure. The water temperature was maintained at 24–25°C. The mice were allowed to swim for 10 min at least 1 day before the normal experiments were performed. Twenty-four hours after drug administration, the mice were placed in a quiet behavioral testing room and left undisturbed for at least 1 h before initiation of the experiment. The behavioral test was performed under subdued light (the same condition controlled during the tail suspension and open field tests). During the experiments, the mice were allowed to swim for 6 min, and their activity was videotaped. The duration of immobility, which was defined as the time during which the mice were floating or remained motionless, was assessed during the last 4 min.

### Tail Suspension Test (TST)

In the TST, the mice were suspended by affixing their tails to the edge of a shelf at a height of 80 cm above the floor, and each animal was subjected to a 6-min suspension session. The activity of the mice was videotaped. The duration of immobility, which was defined as the time during which the mice remained motionless, was assessed during the last 4 min.

### Open Field Test (OFT)

The OFT is a standard method for profiling the exploratory behavior and general activity of mice. The apparatus was composed of opaque black plastic (35 cm × 35 cm × 40 cm) and was placed in a soundproof room. The mice were randomly placed in the center of the field and were allowed to explore the box for 10 min, and their activities were monitored online. The path and velocity of each animal were calculated offline.

### Protein Extraction and Western Blotting

Twenty-four hours after drug administration, the mice were anesthetized with pentobarbital sodium (80 mg/kg, i.p.) and euthanized by decapitation. The bilateral mPFC regions were dissected immediately and homogenized in RIPA Lysis Buffer (Beyotime, P0013B) with 1 mM PMSF (Sigma, P7626), and dissociated by sonic disruption. The homogenate was centrifuged at 14,000 g for 15 min, and the total protein concentration was assessed. The primary and secondary antibodies used in the Western blotting assay were anti-nNOS (1:500, Abcam, ab76067), anti-iNOS (1:500, Abcam, ab15323), anti-BDNF (1:1,000, Abcam, ab108319), anti-β-tubulin (1:1,000, Cell Signaling Technology, 2128), and goat anti-rabbit IgG (1:5,000, Abcam, ab6721) antibodies. An enhanced chemiluminescence (ECL, Thermo, 32109) reaction solution was added. The samples were then recorded on X-ray film (Carestream, 8294985) for visualization, and the immune reactivity was quantified using ImageJ software.

### Electrophysiological Techniques

The mice were anesthetized using pentobarbital sodium (80 mg/kg, i.p.) 24 h after drug administration, and then the electrophysiological experiment was performed. The recording lasted for 3–5 h, and during the recording period, the mice were maintained under anesthesia. The anesthetized mice were then mounted on a stereotaxic apparatus (Narishige, Japan) for extracellular recording. The body temperature was maintained at 36–37°C using a thermostatically controlled heating pad (ATC1000; WPI). In our study, we mainly recorded the spontaneous neuronal activities in the mPFC. The glass electrodes were filled with 2 M NaCl (Sigma, St Louis, MO, USA) and 0.5% Chicago sky blue (Sigma, C8679) solution. The electrodes were placed in the mPFC (coordinates: A/P: + 2.0 to +2.9 mm; M/L: 0 to ±0.90 mm; D/V: -2.5 to -3.5 mm) by using an electric-microdrive (Narishige). Neuronal firing was recorded by a preamplifier and bandpass filtered (Axon clamp, 900A). The data were analyzed with an oscilloscope (Nicolet, Model 2090-I, USA) and stored in a computer equipped with a Clampfit (10.2 Axon, USA) analysis system. To obtain stable firing, the first 2 min of firing were not included in the analysis, and the cells that fired for less than 5 min were excluded. After the recording, the location of the recorded neurons was histologically confirmed in the mPFC.

### Statistics

**Figure 1:**
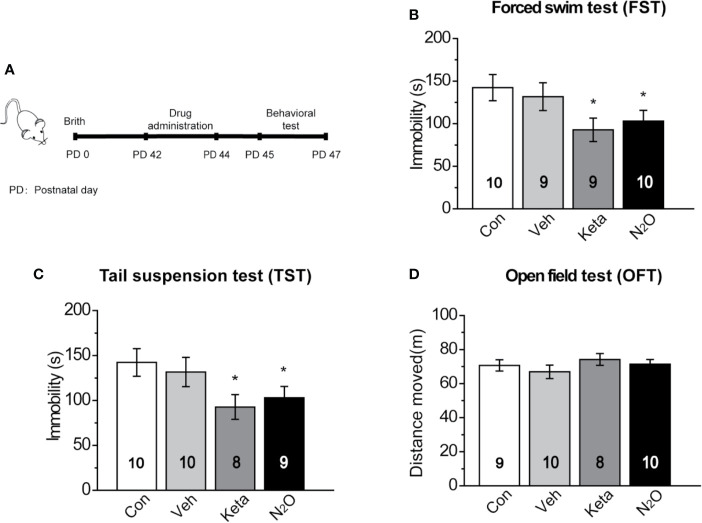
Antidepressant-like effects induced by repeated N_2_O exposure. **(A)** The timeline of the experiments and treatments is presented. Twenty-four hours after the final drug administration, the mice were subjected to forced swim test (FST) **(B)**, tail suspension test (TST) **(C)**, and open field test (OFT) **(D)**. The group details as follows: Control (Con, air exposure); Vehicle (Veh, 50% N_2_ + 50% O_2_ exposure); Ketamine (Keta, 20 mg/kg, i.p.); N_2_O (50% N_2_O + 50% O_2_ exposure). All gas exposures were performed by administering a single dose for 2 h per day for three consecutive days. Ketamine was injected only once at the last day of drug exposure. The immobility time of the mice in the FST and TST was measured, and the total distance that the mice moved in the OFT was measured. The inserted number represents the number of animals in each group. *p < 0.05 compared with the control group.

**Figure 2:**
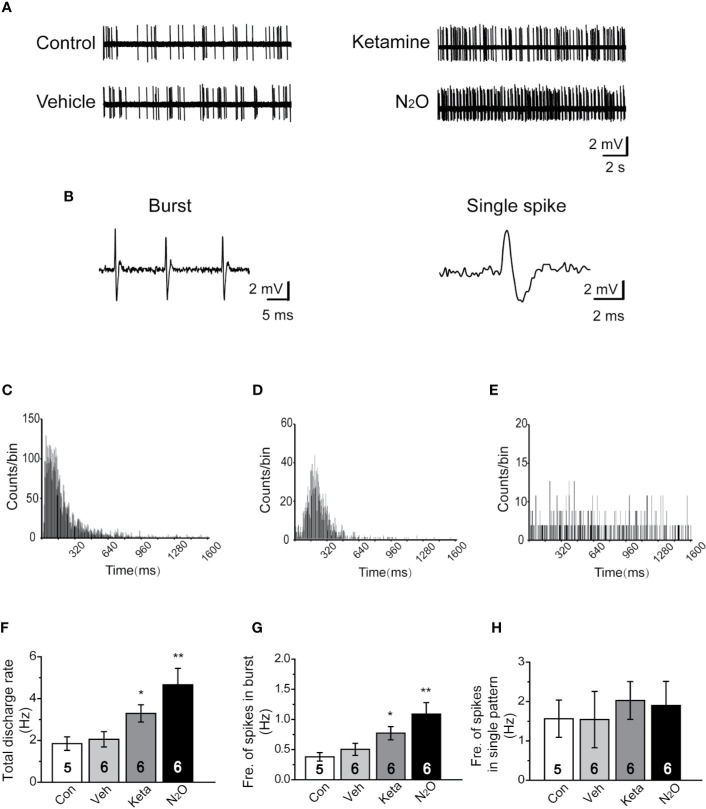
N_2_O enhanced the neuronal activity in the mPFC. In vivo extracellular electrophysiological recordings were performed 24 h after the final drug treatment. **(A)** Representative samples of the spontaneous firing activities of each group are shown. **(B)** Representative traces of the burst firing pattern (left) and single firing pattern (right) are shown. **(C)** The burst firing pattern is represented by a positive-skewed distribution of the inter-spike interval histogram (ISIH). The single firing patterns are represented by a nearly symmetrical ISIH **(D)**, and the other is a relatively straight ISIH **(E)**. The numbers of burst and single spikes were counted in the discharge rate. **(F)** The total discharge rates of the neurons in the mPFC of the different groups were counted. **(G)** The frequency of spikes in the burst firing patterns was shown for the different groups. **(H)** The frequency of spikes in the single firing patterns was shown for the different groups. The inserted number was the number of animals used for the electrophysiological recording. The group info is consistent with that in **Figure 1**. *p < 0.05 and **p < 0.01 compared with the control group.

**Figure 3:**
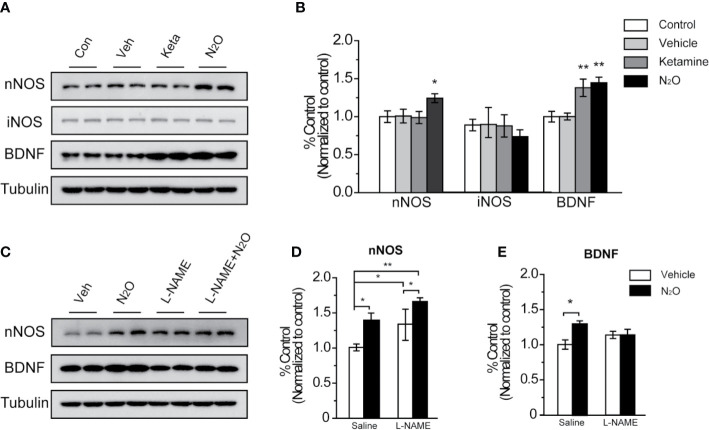
N_2_O enhanced the expression levels of nNOS and BDNF in the mPFC. All the mPFC tissues were extracted 24 h after the final drug administration. **(A**, **C)** Western blot images and **(B, D, E)** quantification analysis of nNOS, iNOS and BDNF expression levels in the mPFC. The β-tubulin was measured as the reference protein. For panels **(A, B)**, the group info is consistent with that in **Figures 1** and **2**. *p < 0.05 and **p < 0.01 compared with the control group. For panels **(C–E)**, the mouse mPFCs were pre-infused with saline (5 μL/side) or L-NAME (5 μg/side) 30 min before every gas exposure. The groups were as follows: Vehicle (saline or L-NAME mPFC infusion before 50% N_2_ + 50% O_2_ exposure) and N_2_O (saline or L-NAME mPFC infusion before 50% N_2_O + 50% O_2_ exposure). *p < 0.05 and **p < 0.01 compared with the vehicle group.

**Figure 4:**
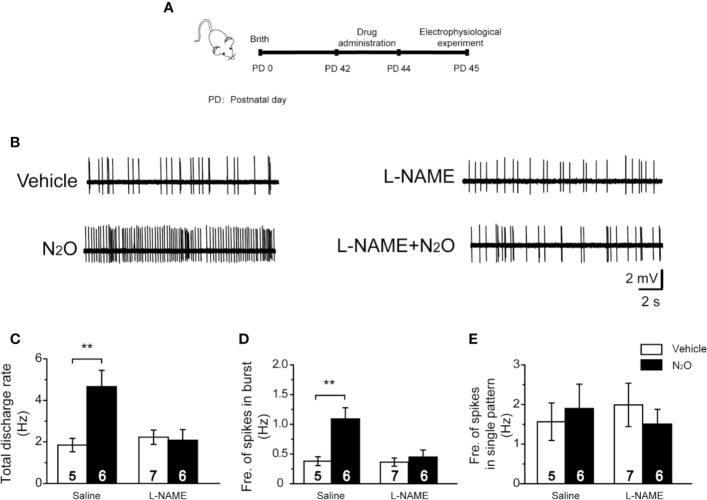
A NOS inhibitor reversed the action of N_2_O on the neuronal activity in the mPFC. **(A)** The timeline of the experimental process. In vivo extracellular electrophysiological recordings were performed 24 h after the final drug treatment. **(B)** Representative samples of the spontaneous firing activities of each group are shown. The numbers of burst and single spikes were both counted in the discharge rate. **(C)** The total discharge rates of the neurons in the mPFC were counted for the different groups. **(D)** The frequency of spikes in the burst firing patterns was shown for the different groups. **(E)** The frequency of spikes in the single firing patterns was shown for the different groups. The mice were pretreated with saline (10 mL/kg, i.p.) or L-NAME (20 mg/kg, *i.p.*) by intraperitoneal injection 30 min before the gas exposure. The groups were as follows: Vehicle (saline or L-NAME intraperitoneal injection before 50% N_2_ + 50% O_2_ exposure); N_2_O (saline or L-NAME intraperitoneal injection before 50% N_2_O + 50% O_2_ exposure). The inserted number was the number of animals used for the electrophysiological recording. **p < 0.01 compared with the vehicle group.

**Figure 5:**
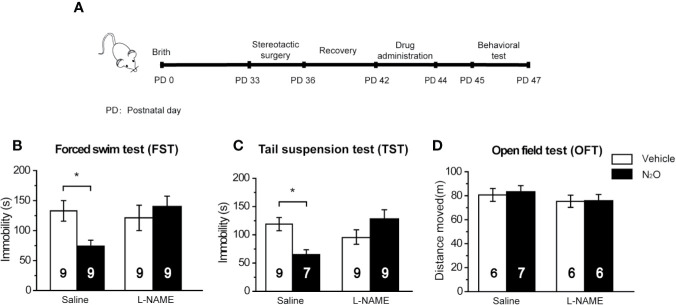
The antidepressant-like effects of N2O were blocked by a NOS inhibitor. **(A)** The timeline of the experimental process. All the experiments were performed 24 h after the final drug administration, and the mice were then subjected to FST **(B)**, TST **(C)** and OFT **(D)**. The mPFC of the mice was pre-infused with saline (5 μL/side) or the NOS inhibitor L-NAME (5 μg/side) 30 min before the gas exposure. The group info is consistent with that in **Figures 3C–E**. The immobility time of the mice in the FST and TST was measured, and the total distance that the mice moved in the OFT was measured. The inserted number represents the number of animals in each group. *p < 0.05 compared with the vehicle group.

The data analysis was performed in double-blinded manner. The data are expressed as the means ± SEMs. SPSS software (version 19 for Windows) was used for the statistical analyses. The statistical parameters, including the exact value of n, precision measures (means ± SEMs) and statistical significance, are presented in the figures. In single drug treatment experimental design (**Figures 1**, **2**, **3A, B**, and **Supplementary Figures 1** and **3**), the data were evaluated by *one-way ANOVA* with a *post hoc least significant difference (LSD)* test. In the experimental design with an added NOS inhibitor (L-NAME, **Figures 3C, D**–**5** and **Supplementary Figure 2**), the data were analyzed using *two -way ANOVA* and *post hoc LSD tests*. *p* < 0.05 indicated statistical significance.

## Results

### Repeated N_2_O Exposure Exerted Antidepressant-Like Effects in Mice

In this study, *50%* N_2_O mixed with 50% O_2_ mixture was used based on the clinical study by Nagele et al. (10). First, we assessed whether N_2_O exerted antidepressant-like effects in the male mice. A single dose of the N_2_O mixture was administered for 2 h per day, and this exposure was repeated on three consecutive days (**Supplementary Figure 1**). To avoid stress response, three behaviors (FST, TST, and OFT) were tested once a day. The FST, TST, and OFT were performed 24, 48, and 72 h after the last exposure to N_2_O, respectively. The timeline of the experiments and treatments is presented in **Figure 1A**. The results showed that both repeated N_2_O exposure, and ketamine treatments significantly reduced the immobility duration in the FST and TST compared with those of the control and vehicle groups [**Figure 1B**, one-way ANOVA, *F (3, 34)* = 2.896, *p* = 0.049; **Figure 1C**, one-way ANOVA, *F (3, 33)* = 2.923, *p* = 0.048]. Total distance in the OFT was not significantly altered in the N_2_O- and ketamine-treated groups [**Figure 1D**, one-way ANOVA, *F (3, 33)* = 0.455, *p* = 0.715], and this finding excluded the possibility that N_2_O or ketamine-induced psychomotor changes and yielded false-positive results. Collectively, the results indicated that repeated exposure to N_2_O induces antidepressant-like responses in mice.

### N_2_O Increased Neuronal Activity in the mPFC

To understand whether N_2_O can affect neuronal activity, the spontaneous neuronal activity was assessed through *in vivo* extracellular electrophysiological recordings. The neural activity of the prelimbic subregion of the mPFC (PrL, A/P: +2.0 to +2.9 mm; M/L: 0 to ± 0.9 mm; D/V: -2.5 to -3.5 mm; **Supplementary Figure 3B**) was recorded. Representative samples of the spontaneous firing activities of different groups are shown (**Figure 2A**). A positive-skewed distribution in the inter-spike interval histogram (ISIH) (**Figure 2C**) indicated a burst firing pattern (**Figure 2B**, left). A single firing pattern was observed as the firing activity of neurons fired in irregular single spikes (**Figure 2B**, right). We recorded two discharge single firing patterns: one pattern was represented by a nearly symmetrical ISIH (**Figure 2D**), and the other pattern was represented by a relatively straight ISIH (**Figure 2E**).

Because neurons often showed burst and single-spike firing activities within a single-unit recording, we calculated the discharge rate, which included the burst and single spikes. The results showed that N_2_O can elicit a significant change in the discharge rate of neurons in the mPFC [**Figure 2F**, one-way ANOVA, *F (3, 64)* = 7.637, *p* = 0.0001]. Besides, the burst activity, including the frequency of spikes in the burst, obtained with N_2_O was higher than that of the vehicle group [**Figure 2G**, one-way ANOVA, *F (3, 41)* =10.192, *p* = 0.00009]. Furthermore, N_2_O significantly increased the overall firing rate and shifted the cumulative frequency of inter-spike intervals (ISIs) toward those observed in a burst pattern. Thus, burst firing in the mPFC might contribute to the antidepressant-like effects of N_2_O. We counted and analyzed the burst firing and single firing patterns, respectively, and found that approximately 65% of the recorded neurons exhibited burst firing and the remaining 35% of neurons showed single firing patterns. However, we found that single firing patterns did not cause significant changes in discharge in the different groups [**Figure 2H**, one-way ANOVA, *F (3, 19)* =0.173, *p* = 0.913]. The increase in burst firing obtained after N_2_O administration made major contributions to the enhancement in neuronal activities in the mPFC, which suggested that the burst firing pattern can be regarded as a predictor of the N_2_O treatment response.

### N_2_O Enhanced the Expression Levels of nNOS and BDNF in the mPFC

To further examine the possible mechanisms underlying the antidepressant-like effects of N_2_O treatment, we assessed the changes in protein expression by Western blotting.

NOS has three isoforms, namely, neuronal NOS (nNOS), inducible NOS (iNOS) and endothelial NOS (eNOS), and functional deficits in nNOS or iNOS had been reported to associated with MDD. However, in our study, we found that repeated N_2_O exposure increased the expression level of nNOS but not iNOS in the mPFC [**Figures 3A, B**, one-way ANOVA, *F (3, 20)* = 5.803, *p* = 0.005 for BDNF, one-way ANOVA, *F (3, 20)* = 3.241, *p* = 0.044 or nNOS; and one-way ANOVA, *F (3,12)* = 1.551, *p* = 0.252 for iNOS]. Furthermore, the level of BDNF, a critical neurotropic factor for neurogenesis and neuroplasticity, was also increased by the administration of N_2_O (**Figures 3A, B**). BDNF plays important an role in neural development and the regulation of synaptic plasticity (23), which is diminished in depressive disorders (24).

To confirm whether BDNF expression is dependent on the activation of nNOS after N_2_O administration, we applied NOS inhibitor, L-NAME (5 μg/side), which we locally infused into the mPFC before drug treatment to specifically block nNOS activity in the mPFC. The results showed that N_2_O exposure didn’t further increase BDNF levels in the mPFC when that region was pre-treated with L-NAME [**Figures 3C–E**, two-way ANOVA, *F (1, 19)* = 3.766, *p* = 0.067 for BDNF, and two-way ANOVA, *F (1, 20)* = 1.325, *p* = 0.263 for nNOS]. In addition, we unexpectedly observed that local L-NAME treatment increased nNOS expression levels without affecting BDNF expression levels (**Figures 3C–E**). Because BDNF is a major growth factor in the brain that might represent a potential therapeutic target in MDD, the increase nNOS activation dependent increase in BDNF might be involved in the antidepressant-like effects of N_2_O.

### N_2_O Increases mPFC Neural Activity Mediated by the Activation of nNOS

The above results demonstrated that repeated N_2_O exposure increased BDNF signaling in a nNOS activation-dependent manner. Therefore, we hypothesized that nNOS might play a key role in the N_2_O-induced enhancement of neuronal activity in the mPFC.

To verify this hypothesis, we injected the mice with L-NAME (20 mg/kg, i.p.) in the mPFC 30 min before N_2_O exposure. Twenty-four hours after the final N_2_O exposure, extracellular electrophysiologyical recording was performed in the mPFC (**Figure 4A**). Interestingly, we found that the systemic injection of L-NAME could block the antidepressant-like effects induced by repeated N_2_O exposure [**Supplementary Figure 2**, two-way ANOVA, *F (1, 35)* = 3.259, *p* = 0.080 for FST; two-way ANOVA, *F (1, 33)* = 2.983, *p* = 0.93 for TST; two-way ANOVA, *F (1,30)* = 1.867, *p* = 0.182 for OFT]. We further examined whether mPFC neuronal activity could also be inhibited by pretreatment with L-NAME. As expected, the N_2_O-induced increases in the discharge rate and burst firing were blocked by pretreatment with L-NAME [**Figures 4B, C**, two-way ANOVA, *F (1, 42)* = 3.902, *p* = 0.055; **Figure 4D**; two-way ANOVA, *F (1, 42)* = 3.434, *p* = 0.071]. The irregular firing did not significantly change after drug administration [**Figure 4E**, two-way ANOVA, *F (1, 24)* = 1.485, *p* = 0.235], as described above. These results suggested that the antidepressant-like effects of N_2_O might be due to increased mPFC excitability followed by nNOS activation.

### The Antidepressant-Like Effects of N_2_O Were Dependent on NOS Activation in the mPFC

Based on the above results, we confirmed that N_2_O increased neuronal activity and BDNF expression in the mPFC, and these processes were dependent on the activation of nNOS. Therefore, we further asked whether the antidepressant-like effects of N_2_O were dependent on nNOS activation in the mPFC. To block the activity of nNOS in the mPFC, we pretreated the mPFC with L-NAME 30 min before N_2_O exposure. The behavioral tests were performed 24 h after the final exposure of N_2_O (**Figure 5A**). The results showed that repeated N_2_O exposure decreased immobility duration in the FST and TST was reversed by pretreatment with L-NAME (5 μg/side) in the mPFC (PrL). L-NAME alone showed no significant effects on the FST and TST [**Figure 5B**, two-way ANOVA, *F (1, 32)* = 1.768, *p* = 0.193; **Figure 5C**, two-way ANOVA, *F (1, 29)* = 0.391, *p* = 0.537]. The total distance traveled in the open field did not show significant changes between the groups, which indicated that L-NAME and/or N_2_O exposure did not affect the locomotor activity of the mice [**Figure 5D**, two-way ANOVA, *F (1, 21)* = 0.092, *p* = 0.765]. In summary, these results suggested that the antidepressant-like effects induced by repeated N_2_O exposure were dependent on the activation of nNOS in the mPFC (PrL).

## Discussion

In our study, we investigated the antidepressant-like properties of N_2_O through multiple behavioral tests. To address the underlying mechanism, we explored the neuronal activities in the mPFC and intracellular signal transduction pathways. The results showed that repeated N_2_O exposure increased the neuronal burst-firing activity and upregulated the nNOS and BDNF expression levels in the mPFC. Furthermore, the antidepressant-like effects and enhanced neuronal activities induced by N_2_O were blocked by the inhibition of nNOS. The results suggested that repeated N_2_O exposure enhances BDNF expression level and burst firing rate in an nNOS activation-dependent manner in the mPFC, and these effects might underpin the molecular mechanisms underlying the antidepressant-like effects of N_2_O.

The concentration of general anesthetics is a critical impact factor for CNS function, and the affected sites of the CNS by general anesthetics are concentration dependent (25). The binding preference of general anesthetics for subunits of excitatory and inhibitory neurotransmitter receptors, such as NMDA receptors and GABA_A_ receptors, is also concentration dependent (26). In addition, substantial studies have shown that subanesthetic concentrations prompt neuronal stem cell survival and neurogenesis, but high concentrations may cause neurotoxicity in the CNS (27). For N_2_O, a clinical study by Nagele and colleagues applied short-term subanesthetic N_2_O exposure (50% N_2_O + 50% O_2_ inhaled for 1 h in a single session), which showed antidepressant effects in TRMD. The subanesthetic dose of N_2_O was selected based on the routine use for analgesia and mild sedation in anesthesiology and dentistry (10, 28). In the present study, the 50% N_2_O concentration was selected, but unlike appearances in humans, repeated 2 h rather than a single 1-h subanesthetic N_2_O exposure triggered antidepressant-like effects in mice. However, the underlying mechanism of this phenomenon is unclear. The optimal antidepressant dose of N2O should be further addressed in future studies.

NO is a gaseous neurotransmitter that governs multiple physiological functions in the CNS. NO is synthesized by NOS, which has three isoforms, namely, nNOS, iNOS, and eNOS, and all three NOS isoforms exist in the CNS and affect cell signaling in brain (29). Evidence has shown that deficits in NOS activity are associated with the neurobiology of MDD (30, 31). Especially for nNOS, studies have shown that dysfunction of nNOS in the paraventricular hypothalamic nucleus, locus coeruleus, mPFC, and hippocampus, is related to MDD (29, 32, 33). Besides, iNOS also is involved in stress-triggered depression, as iNOS derived NO and iNOS mRNA levels increased in the cortices of depression animal model (34). However, a lower number of studies reported the relationship between eNOS and depression and genetic studies suggested there is no correlation between eNOS and MDD (35). In the present study, we demonstrated that N_2_O exposure significantly increased the expression level of nNOS rather than iNOS. As the major route for the production of NO in the brain is mediated by nNOS catalysis (29), our results suggest that nNOS might be a dominant molecule in mediating the antidepressant-like effects of N_2_O.

Although abundant reports have emphasized the critical role of nNOS in the neuropathology of MDD, the conclusion is controversial (29). Some studies pointed to an increased activity of nNOS in the corticolimbic system in MDD, and NOS inhibitors showed antidepressant effects through indirect regulation of the monoaminergic system (29, 36). However, several other studies showed that stress exposure diminished nNOS activity in the hippocampus which further caused a deficit in learning and memory processes in animals (37, 38). Postmortem studies also revealed a decrease in nNOS expression in the anterior cingulate cortex (ACC) of MDD patients, which indicated weakened nNOS activity in the process of MDD (39). Therefore, there exists an imbalance but not simply an increase or decrease in nNOS activity in the pathophysiology of MDD. In the present study, we unexpectedly observed that local NOS inhibitor (L-NAME) treatment increased nNOS expression levels without affecting BDNF levels in the mPFC or the depressive behaviors of animals. For this paradoxical phenomenon, we infer that there may exist a feedback mechanism by which L-NAME, as a NOS inhibitor, inhibits the activity of nNOS and further elevates the expression level of nNOS as a result. Because the real activity of nNOS was low (inhibited by L-NAME), L-NAME induced higher expression levels of nNOS could not further activate BDNF signaling and trigger antidepressant-like effects. The concrete mechanisms need to be studied in the future.

BDNF is a developmentally expressed growth factor that regulates plasticity in the adult brain. Preclinical studies have shown that the function of BDNF could be decreased by several forms of stresses Chronic treatments with antidepressants activate BDNF-mediated signaling (40). nNOS-derived NO could modulate BDNF signaling in stress adaptation, which further affects synaptic plasticity in emotionally related brain regions such as cerebral cortex, amygdala, hippocampus and striatum (29, 41). However, the interplay between NO and BDNF signaling has been elusive. Some reports point to that NO appears to negatively modulate BDNF function, because BDNF signaling is augmented by the NOS inhibitor L-NAME (42). However, evidence from cultured hippocampal neurons demonstrated that endogenous low levels of NO could facilitate BDNF signaling, indicating that BDNF signaling is regulated by NO in a concentration-dependent manner (43). In the present study, we observed that the BDNF expression level in the mPFC was upregulated by N_2_O exposure, and the increase of BDNF was blocked by pre-administration of the NOS inhibitor L-NAME. Based on the above information, we suggest that N_2_O exposure with a subanesthetic dose might increase intracellular NO, which activates BDNF-dependent synaptic plasticity. Our findings enhance the understanding of the role of nNOS activation induced increase of BDNF signaling in the antidepressant-like responses and support a promising research direction of N_2_O as a potential antidepressant for clinical use.

Although N_2_O and ketamine have similar antidepressant actions and mechanisms, the antidepressant efficacy of N_2_O might not be robust as that of ketamine. Moreover, previous studies have indicated that N_2_O exposure is associated with many unfavorable side-effects (e.g., euphoria, altered body awareness and image, altered time and perception, and dreamy, detached experiential states) (44) and prolonged N_2_O administration interferes with the activity of vitamin B_12_, which results in sensory ataxia problems (11, 45). Therefore, the efficacy, safety and tolerability of N_2_O should be carefully evaluated in the future. More work needs to be done to investigate the effective initial dose and maintenance dose, the therapeutic drug duration and the potential risks of N_2_O when given repeatedly over time (44).

A valid animal model of depression is necessary to identify the biological mechanisms of MDD and the features of antidepressants. Currently, stress-induced models are the most commonly used depression model, which might be based on the fact that initial episodes of depression are precipitated by adversity. In addition, the etiology-based animal model of depression that directly targeting the underlying biological causations, including the hypothalamus-pituitary-adrenal (HPA) axis, the neuroinflammation system and the neurotransmission, also recapitulating specific dysfunctions of depression (46). MDD is a heterogeneous disease, and diverse animal models are believed to recapitulate different aspects of symptomatic dimensions and neuropathology associated with depression (47). The antidepressant efficacies and pharmacology mechanisms of N_2_O should be carefully detected in the future study.

Mood disorders are characterized by profound deficits in reward circuits of brain. Mesocorticolimbic dopaminergic system, as the most important reward circuits, is constructed by the ventral tegmental area (VTA) originated dopaminergic projections to many brain areas such as the mPFC, nucleus accumbens (NAc), amygdala, and hippocampus. Through highly complex inter-projections, these brain regions have been shown to be involved in emotion-related behaviors, especially in the encoding of stressful events (48). The antidepressants might have broad influences on the reward circuit system. According to literatures, ketamine rapidly increases the activity of reward-related brain regions, including the orbitofrontal cortex, ventral striatum, VTA, amygdala, insula, and the ACC, which accompanied with the fast remission of depressive symptoms by ketamine (49). Moreover, ketamine also have positive impacts on dopaminergic transmission (50, 51). In the present study, we depicted a specific antidepressant mechanism of N_2_O by focusing on the mPFC. We believe that N_2_O might also have diverse influences on other reward-related brain regions that similar to the effects of ketamine, which should be addressed in the future studies.

In conclusion, we verified that repeated N_2_O exposure exerts antidepressant-like effects in animals. Our preliminary results suggested that repeated N_2_O exposure can enhance BDNF expression level and burst firing rates in an nNOS activation dependent manner in the mPFC, which might underpin the pharmacological mechanism underlying the antidepressant-like effects of N_2_O exposure. Collectively, the results obtained in the present study might provide some theoretical basis for developing a relatively promising and safe method for the treatment of MDD.

## Data Availability Statement

The datasets generated for this study are available on request to the corresponding authors.

## Ethics Statement

The animal study was reviewed and approved by All the animal studies and experimental procedures were approved by the Animal Care Committees of the Shanghai Institute of Materia Medica, Chinese Academy of Sciences, and the experiments were performed in accordance with EU Directive 2010/63/EU on the protection of animals used for scientific purposes.

## Author Contributions

WL, QL, and BY conducted the majority of the experiments, including stereotactic surgery, electrophysiological part and behavioral tests. HC, JL, and WD conducted the western blotting and stereotactic surgery. ZX, JL, and FS carried out the animal behavioral tests and electrophysiological part. TL and FG conducted the data analysis and brain regions detection. WL, QL, BY, BZ, and ZL. prepared the manuscript. WL, BZ, and ZL conceived and supervised the project. All authors contributed to the article and approved the submitted version.

## Funding

This work was supported by the National Natural Science Foundation (grant numbers 81771188, 31671049, 81971418, and 81901376), Science and Technology Commission of Shanghai Municipality (grant numbers 14411966700 and 16411967400), the China Postdoctoral Science Foundation Funded Project (grant number 2017M621535), the Ministry of Science and Technology (grant number 2013CB91060101), Shanghai Municipal Commission of Health and Family Planning (grant number 201740072), the Fundamental Research Funds for the Central Universities (grant number 22120180534), and “Personalized Medicines-Molecular Signature-based Drug Discovery and Development”, Strategic Priority Research Program of the Chinese Academy of Sciences (grant numbers XDA12040302 and XDA120402140).

## Conflict of Interest

The authors declare that the research was conducted in the absence of any commercial or financial relationships that could be construed as a potential conflict of interest.
