# Supplementary material for: Repeated Nitrous Oxide Exposure Exerts Antidepressant-Like Effects Through Neuronal Nitric Oxide Synthase Activation in the Medial Prefrontal Cortex
Source: Front Psychiatry. 2020 Sep 3;11:837. doi: 10.3389/fpsyt.2020.00837 (PMC7495238; doi:10.3389/fpsyt.2020.00837)
Supplement: Supplementary file 1 [file Table_1.docx]

**Supplementary Materials**

**Repeated nitrous oxide exposure exerts**

**antidepressant-like effects through neuronal nitric oxide**

**synthase activation in the medial prefrontal cortex**

Wei Liu*^1+^*, Qian Li*^1+^*, Binglu Ye*^1+^*, Hang Cao*^1^*, Fuyi Shen*^1^*, Zhendong Xu*^1^*, Weijia Du*^1^*, Fei Guo*^2^*, Jinqi Liu*^5^*, Tianyu Li*^2^*, Bing Zhang*^1,3*^* and Zhiqiang Liu*^1,4*^*

**^1^** Shanghai First Maternity and Infant Hospital, Tongji University School of Medicine, Shanghai, 201204, China.

**^2^** Key Laboratory of Receptor Research, Shanghai Institute of Materia Medica,

Chinese Academy of Sciences, Shanghai, 201203, China.

**^3^** Clinical and Translational Research Center, Shanghai First Maternity and Infant Hospital, Tongji University School of Medicine, Shanghai, 201204, China.

**^4^** Anesthesia and Brain Function Research Institute, Tongji University School of

Medicine, Shanghai, 200082, China.

5, The MacDuffie School, 66 School Street, Granby, MA 01033, USA.

*^⁎^* Corresponding authors at: No.2699 Gaoke West Road, Pudong New District,

Shanghai, 201204, China.

E-mail addresses: drliuzhiqiang@163.com (Z.Q. Liu), bingozzz@126.com (B. Zhang)

*^+^* These authors contributed equally to this work.

**1. Methods**

**1.1 Gas exposure**

In the present study, we payed closely attention to the dose of N_2_O, we selected 50% N_2_O which was based on the routine use for analgesia and mild sedation in anesthesiology and dentistry. For antidepressant dose exploration of N2O, we tried 1 hour or 2 hours with single or 2-3 sessions N_2_O exposure. The mice were placed in a transparent and temporary airtight chamber (35 × 25 × 15 cm) at room temperature for 10 min of acclimatization prior to the gas exposure sessions. The concentration of N_2_O was monitored in real time. The gas flow was maintained at 2-3 L/min. At the end of the drug administration period, the mice were returned to their home cages.

**2. Supplementary figures and figure legends**

***Supplementary Figure 1***

***

***

***The dose exploration for the antidepressant-like effects induced by N_2_O.***

The differences in the duration of a single dose (hour) and the frequency of administration (day) used in the behavioral tests are shown in **Sup. 1**(one-way ANOVA, *F (3,34)* = 2.730, *p* = 0.183 for **A**; one-way ANOVA, *F (3,36)* = 0.624, *p* = 0.604 for **B**; one-way ANOVA, *F (3,28)* = 1,727, *p* = 0.048 for **C**; one-way ANOVA, *F (3,24)* = 0.575, *p* = 0.349 for **D**). 24 h after drug administration, the mice were analyzed using forced swim test (FST) and open field test (OFT). The immobility time of the mice in the FST were measured, and the total distance that the mice moved in the OFT was measured. For 1-hour/session (**A and B**) and 2-hour/session (**C and D**) the mice were divided into the following groups: the mice in the vehicle group were subjected to 1/2-h exposure to 50% N_2_ + 50% O_2_ for 3 day, the mice in the 1-Day group were subjected to 1/2-h exposures to 50% N_2_O + 50% O_2_ for 1 day, the mice in the 2-Day group were subjected to 1/2-h exposures to 50% N_2_O + 50% O_2_ for 2 day and the mice in the 3-Day group were subjected to 1/2-h exposure to 50% N_2_O + 50% O_2_ for 3 day. The inserted number represent the number of animals used in each group. **p* < 0.05 compared with the vehicle group.

***Supplementary Figure 2***

***

***

**The antidepressant-like effects of N_2_O were blocked by the intraperitoneal injection of L-NAME**. All the experiments were performed 24 h after the final drug administration, and the mice were then subjected to FST **(A,** two-way ANOVA, *F (1,35)* = 3.259, *p* = 0.080**)**, TST **(B,** two-way ANOVA, *F (1,33)* = 2.983, *p* = 0.93**)** and OFT **(C,** two-way ANOVA, *F (1,30)* = 1.867, *p* = 0.182). The mice were pretreated with saline (10 mL/kg, i.p.) or L-NAME (20 mg/kg, i.p.) 30 min before the ventilation operations. The groups as following: Vehicle (saline or L-NAME intraperitoneal injection before 50% N_2_ + 50% O_2_ exposure); N_2_O (saline or L-NAME intraperitoneal injection before 50% N_2_O + 50% O_2_ exposure). All the gas exposures were performed by administering a single dose for 2 h per day for three consecutive days. The immobility time of the mice in the FST was measured, and the total distance that the mice moved in the OFT was measured. The inserted number represent the number of animals used in each group. **p* < 0.05 compared with the vehicle group.

***Supplementary Figure 3***


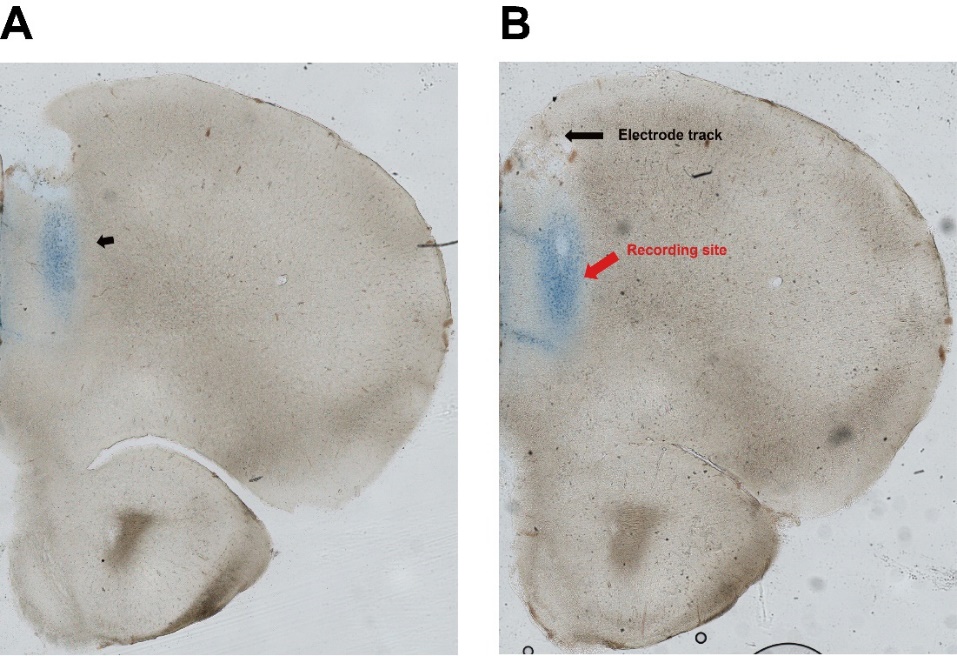


**The visualization of mPFC brain local injection site (A) and the in vivo electrophysiological recording site (B). (A)**The schematic diagram of mPFC local injection, the black arrow indicates the drug diffusion region in the mPFC. **(B)** Image showing the recording site (red arrow) and electrode entry track (black arrows) in the mPFC. The results showed that the local injection site and the recording site were both located at the prelimbic subregion of mPFC (PrL).
